# Seasonality of Influenza and Optimizing Timing of Vaccination: Systematic Review and Meta-Analysis

**DOI:** 10.7759/cureus.93607

**Published:** 2025-09-30

**Authors:** Abhishek Padhi, Pritesh Bhatt, Jagruti Chauhan, Bhoomika Rajyaguru, Stuti Agarwal, Anil Chaudhary, Mayuri Bhise, Ashwini Agarwal

**Affiliations:** 1 Microbiology, All India Institute of Medical Sciences, Rajkot, IND; 2 Virology, All India Institute of Medical Sciences, Rajkot, IND; 3 General Medicine, Armed Forces Medical College, Pune, IND

**Keywords:** india, influenza, seasonality, vaccination timing, vaccine effectiveness

## Abstract

Influenza continues to be a major global public health concern, with substantial regional variability in its seasonality and vaccine effectiveness. While temperate regions experience predictable winter peaks, tropical and subtropical countries exhibit complex patterns, including monsoon-related and year-round activity. This variability poses challenges to the current vaccination strategies, which are often guided by broad hemispheric recommendations rather than localized epidemiological data.

This systematic review and meta-analysis aimed to assess global influenza seasonality, evaluate the timing of vaccination programs, and explore the impact of misalignment on vaccine effectiveness (VE), with a particular focus on India. We searched five databases (PubMed, Scopus, Medline, Google Scholar, and Cochrane Library), and a total of 32 studies were included in the qualitative synthesis, with 12 eligible for meta-analysis. The pooled VE across studies was 27.22% (95% CI: 17.36-37.08%), with significant heterogeneity (I² = 93.2%, p < 0.001). While modest, this level of protection still averts cases when coverage is high, and the marked heterogeneity (I² = 93.2%) signals that timing, age mix, and strain match substantially influence real-world impact. The quality of the included studies was assessed with the Cochrane Risk of Bias tool 2.0 and the Newcastle-Ottawa Scale.

The meta-regression identified age and timing of vaccination as key contributors to variability in vaccine effectiveness. India exemplifies the challenge of heterogeneity within a single country. Northern regions such as Jammu and Kashmir display winter peaks (December-March), while central and eastern cities, including Delhi, Lucknow, and Kolkata, experience monsoon-season peaks (July-September), and South Indian cities such as Chennai and Vellore peak influenza activity during the late monsoon (October-November). However, influenza vaccination in India is delivered primarily through private sector providers, often beginning in April, and is not part of a centrally coordinated campaign, which may not align with regional peaks. This misalignment likely contributes to suboptimal VE in several regions. The findings underscore the urgent need to re-evaluate vaccination strategies globally and within India. Adoption of region-specific vaccination schedules - tailored to local influenza seasonality, evidence-informed, and supported by robust surveillance accounting for climate, geography, and population dynamics - could significantly enhance vaccine performance and reduce disease burden.

This study is registered with PROSPERO, CRD42024611680.

## Introduction and background

Influenza remains a significant global public health concern, contributing substantially to morbidity and mortality annually. The World Health Organization (WHO) estimates that annual influenza epidemics result in approximately 3 to 5 million cases of severe illness and between 290,000 and 650,000 respiratory-related deaths worldwide [1]. Despite the availability of vaccines, influenza continues to impose a considerable burden on healthcare systems, economies, and societies globally [2]. However, regional surveillance quality varies widely, with many tropical and subtropical regions lacking robust epidemiological data needed to inform vaccination strategies, as demonstrated in a systematic review of influenza surveillance in the Eastern Mediterranean and North African region [3].

Vaccination is the primary strategy for preventing influenza and its associated complications. However, influenza vaccine effectiveness (VE) can vary significantly each year, influenced by factors such as the match between vaccine strains and circulating viruses, age and health status of recipients, and timing of vaccine administration relative to the influenza season [4]. A U.S. Flu VE Network analysis across seven seasons found that VE against influenza A(H3N2), A(H1N1)pdm09, and B/Yamagata hospitalizations decreased with increasing time since vaccination, with an average decline in VE of about 8-9% per month postvaccination, indicating significant intraseason waning of inactivated vaccine protection and the need to reassess vaccination timing strategies [5]. Studies have consistently shown VE ranges from 40-60% during seasons with a good antigenic match between vaccine strains and circulating influenza viruses [6]. Conversely, mismatches significantly reduce VE, highlighting the critical importance of accurate strain selection and optimal vaccination timing [4]. A pediatric test-negative study in Hong Kong showed VE dropped from 79% (95% CI: 64-88) at 0.5-2 months post-vaccination to 45% (22-61) at 6-9 months, with a monthly loss of 2-5%, illustrating both waning and mismatch effects on protection [7].

The timing of influenza vaccination is essential to ensure optimal protection [3]. In temperate regions, influenza activity typically peaks during winter months, prompting vaccination campaigns in the autumn [8]. In contrast, influenza seasonality in tropical and subtropical regions is less predictable, with peaks occurring variably throughout the year or even exhibiting year-round low-level circulation [8, 9]. Ecological time-series data from Hangzhou, China, linked PM₂.₅, PM₁₀, NO₂, and SO₂ to increased influenza-like illness risks, especially in cold seasons, highlighting air pollution as a non-viral driver of influenza epidemiology [10]. For example, studies from tropical Asia have demonstrated that influenza activity commonly peaks between May and October, differing markedly from typical winter peaks observed in temperate climates [11]. This variability complicates the establishment of optimal vaccination timing strategies in these regions.

The WHO currently recommends biannual updates to influenza vaccine compositions, one for each hemisphere, based on global surveillance data [12]. Recent evidence indicates significant intraseasonal waning of vaccine‐induced immunity, with antibody titres declining markedly within six months post‐vaccination, underscoring the potential need for booster dosing, staggered regional rollouts, or shifting campaign start dates to better match local peaks [13]. Nevertheless, this approach might inadequately address the distinctive influenza seasonality patterns observed in equatorial regions [14]. For instance, studies in countries near the equator, such as Bangladesh, have shown influenza circulation patterns more closely resembling southern hemisphere trends, despite their geographic position in the northern hemisphere [15]. This disparity indicates the inadequacy of traditional hemispheric guidelines and underscores the need for tailored strategies. An analysis of global vital-record and surveillance data estimated 291,243-645,832 annual influenza-associated respiratory deaths, with the highest rates in Southeast Asia and sub-Saharan Africa, reinforcing the urgent need for region-specific vaccination policies [16].

In India, influenza seasonality varies significantly across regions, with some areas experiencing peaks during the monsoon season (July-September), while others observe peaks during winter months. However, influenza vaccines in India are not distributed via a formal government campaign but rather through private channels, usually offered from April onward, which can misalign with the actual timing of regional influenza activity. Studies investigating influenza seasonality across Indian regions have emphasized the importance of developing region-specific vaccination strategies to optimize public health outcomes [9, 17].

Recognizing these challenges, there is a pressing need to reevaluate and optimize influenza vaccination strategies globally. Effective vaccination requires careful consideration of local epidemiological patterns, seasonality, and viral strain variations [4, 18]. Current evidence supports region-specific adjustments to vaccination schedules rather than a universal, hemispheric-based approach [8, 9]. Consequently, aligning vaccination campaigns more closely with local epidemiological realities could significantly enhance their public health impact [19].

Given the complexities of global influenza seasonality and VE, this systematic review and meta-analysis aim to address key questions such as "What are the global patterns of influenza seasonality?", "How effective is vaccination by region and age?", and "Can vaccination timing be optimized for tropical settings?" By synthesizing available data from multiple studies, this review seeks to inform evidence-based public health policies and develop tailored vaccination strategies. Ultimately, our goal is to provide insights that enable public health officials worldwide to enhance the effectiveness of influenza prevention efforts through optimized vaccine timing and administration.

## Review

Methodology

Protocol and Registration

This systematic review and meta-analysis were conducted per Preferred Reporting Items for Systematic Reviews and Meta-Analyses (PRISMA) 2020 guidelines (See Table A1 for the PRISMA 2020 checklist) [20]. The study protocol was registered with the International Prospective Register of Systematic Reviews (PROSPERO) under the registration number CRD4202461168.

Eligibility Criteria

Studies published in English between January 1, 2014, and November 30, 2024, were included in the study. This 10‑year window captures changes in vaccine composition, post‑pandemic surveillance enhancements, and recent temperate vs tropical data. 

Additionally, those focusing on human influenza, addressing aspects such as seasonality, vaccination timing, vaccine effectiveness, age-related factors, geographical variations, climatic influences, and circulating virus strains, were also included.

Furthermore, study designs included randomized controlled trials (RCTs), observational studies, multicenter studies, controlled clinical trials, comparative studies, clinical studies, pragmatic clinical trials, and validation studies. RCTs contributed vaccine efficacy data where VE was directly measured.

Exclusion criteria encompassed studies lacking relevant data on influenza seasonality or vaccination timing, non-human studies, reviews, editorials, and case reports. Studies limited to strain surveillance or outbreak response without data on vaccination timing or VE were also excluded.

Information Sources and Search Strategy

A comprehensive literature search was conducted across multiple databases: PubMed, Google Scholar, Medline, Scopus, and the Cochrane Library. The search spanned publications from January 1, 2014, to November 30, 2024.​

The search strategy incorporated Medical Subject Headings (MeSH) and relevant keywords, as can be seen in Table 1. The detailed search strategy is available upon request.​

**Table 1:** Search strategy

Search strategy	
MeSH and keywords	(("Influenza, Human"[MeSH Terms] OR "influenza" OR "flu") AND (("Seasonal Variation"[MeSH Terms] OR "seasonality" OR "flu season" OR "epidemic peaks") OR ("Vaccination"[MeSH Terms] OR "Influenza Vaccines"[MeSH Terms] OR "vaccine timing" OR "vaccination strategies")) AND (("Age Factors"[MeSH Terms] OR "age groups" OR "children" OR "adults" OR "elderly") OR ("Geography"[MeSH Terms] OR "geographical regions" OR "tropical" OR "temperate" OR "subtropical") OR ("Climate"[MeSH Terms] OR "Temperature"[MeSH Terms] OR "Humidity"[MeSH Terms] OR "climatic factors") OR ("Influenza A Virus"[MeSH Terms] OR "Influenza B Virus"[MeSH Terms] OR "virus strains" OR "H1N1" OR "H3N2" OR "B/Victoria" OR "B/Yamagata")))

Study Selection

The study selection process involved three reviewers: PB, JC, and AP. Initially, PB and JC independently screened the titles and abstracts of all retrieved articles to identify potentially eligible studies. Subsequently, full-text articles of selected studies were assessed for eligibility based on the predefined inclusion and exclusion criteria. Discrepancies between PB and JC were initially resolved through discussion between them and with AC and MB. In cases where consensus could not be reached, AP acted as an independent adjudicator to make the final decision, ensuring unbiased selection.​

Data Extraction

Data extraction was performed independently by PB and JC using a standardized data extraction form. The following information was extracted: study characteristics (including author(s), publication year, country of study, study design, and sample size); participant characteristics (age groups only, as gender data were inconsistently reported and thus not analyzed); outcomes (e.g. timing of influenza peaks, vaccination timing, vaccine effectiveness, and circulating influenza strains); and statistical data (effect sizes, confidence intervals, and measures of heterogeneity).

Any discrepancies in data extraction were discussed between PB and JC, with AP consulted for resolution when necessary.​

Quality Assessment

The methodological quality of included studies was assessed independently by PB and JC. For randomized controlled trials, we utilized the Cochrane Risk of Bias Tool V2.0 [21]. Observational studies were evaluated using the Newcastle-Ottawa Scale (NOS) [22]. Disagreements in quality assessments were resolved through discussion or consultation with AP.

Data Synthesis and Statistical Analysis

A meta-analysis was conducted using a random-effects model to account for potential heterogeneity among studies [23]. All statistical analyses - including pooled effect estimation and forest-plot generation - were performed in R version 4.4.0 using the meta, metafor, and forestplot packages. The primary outcome was the pooled VE across different regions and age groups. Heterogeneity was assessed using Cochran's Q test and quantified with the I² statistic, with values of 25%, 50%, and 75% representing low, moderate, and high heterogeneity, respectively [24].

Meta-regression analyses were performed to explore potential sources of heterogeneity, including age groups, geographical regions, and timing of vaccination. The adequacy of these models was assessed through an examination of the regression outputs and consistency of findings across subgroups, and no single study was found to unduly influence the results. Results estimates were presented with corresponding 95% confidence intervals.

Reporting

The findings of this systematic review and meta-analysis are reported in accordance with the PRISMA 2020 guidelines. A PRISMA flow diagram illustrating the study selection process is included in the results section.​

Results

The flow diagram summarizes the study identification, screening, eligibility assessment, and inclusion process as per PRISMA 2020 guidelines. A total of 6,538 records were identified: 4,631 through database searches (PubMed, Scopus, Medline, Google Scholar, Cochrane) and an additional 1,907 from a focused MeSH search in PubMed. After merging and removing duplicates, 5,976 unique records remained for screening.

**Figure 1:**
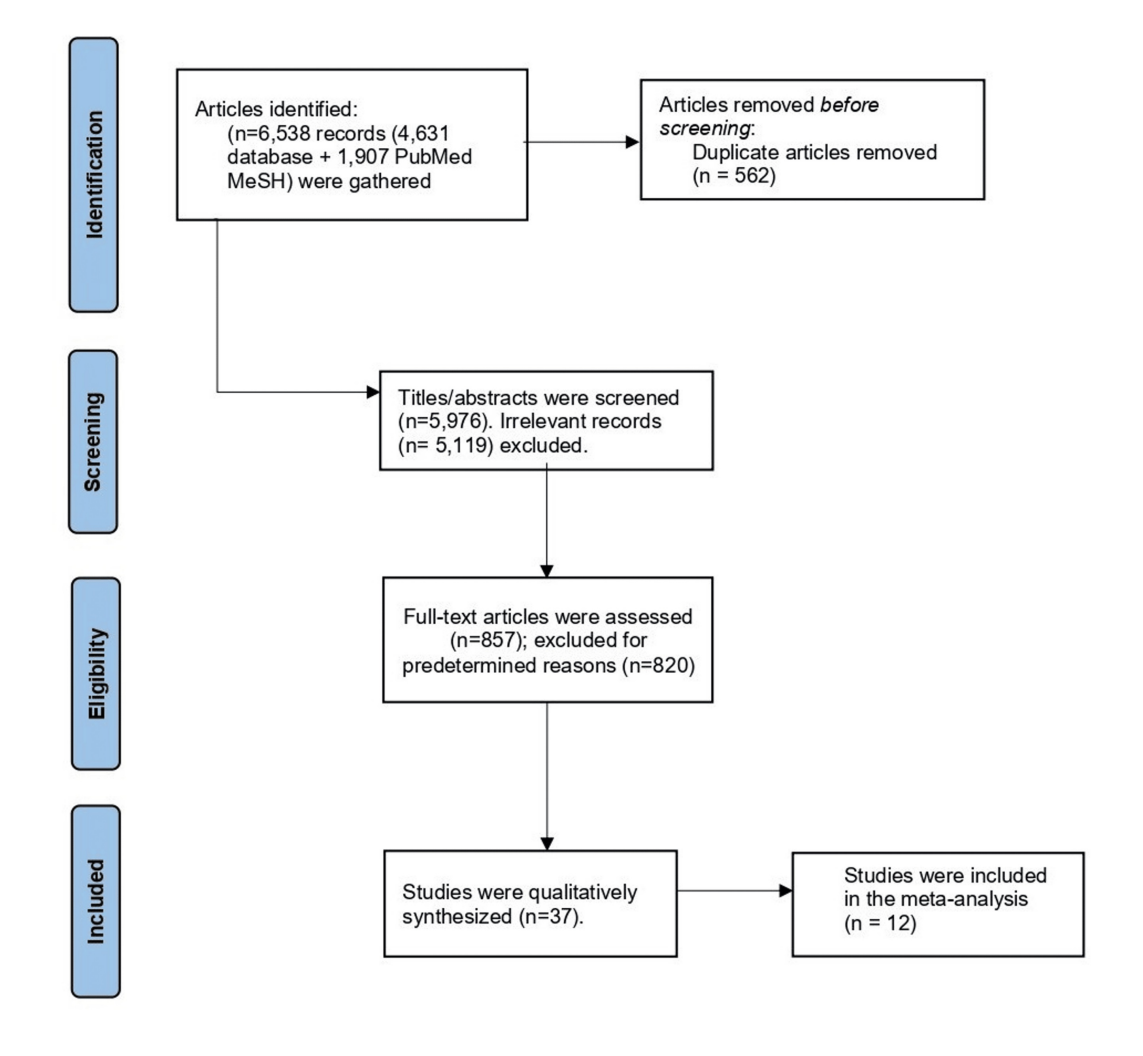
PRISMA 2020 Flow Diagram Showing Study Selection Process Source: PRISMA 2020 statement [20]. Exclusion criteria encompassed studies lacking relevant data on influenza seasonality or vaccination timing, non-human studies, reviews, editorials, and case reports. Studies limited to strain surveillance or outbreak response without data on vaccination timing or vaccine effectiveness were also excluded.

Following initial screening, 857 unique full-text articles were assessed for eligibility. Of these, 820 were excluded due to one or more of the following reasons: irrelevance to influenza seasonality or vaccination timing, insufficient data on VE, or non-human or non-original research articles (e.g., editorials, reviews). A total of 37 studies met all eligibility criteria and were included in the systematic review. Out of these, 12 studies provided adequate quantitative data and methodological quality for inclusion in the meta-analysis. These 12 studies were subjected to pooled analysis for estimating overall VE and were included in further subgroup and meta-regression analyses. This flowchart ensures transparency and reproducibility of the review process and aligns with international reporting standards (Figure 1) [20].

**Figure 2:**
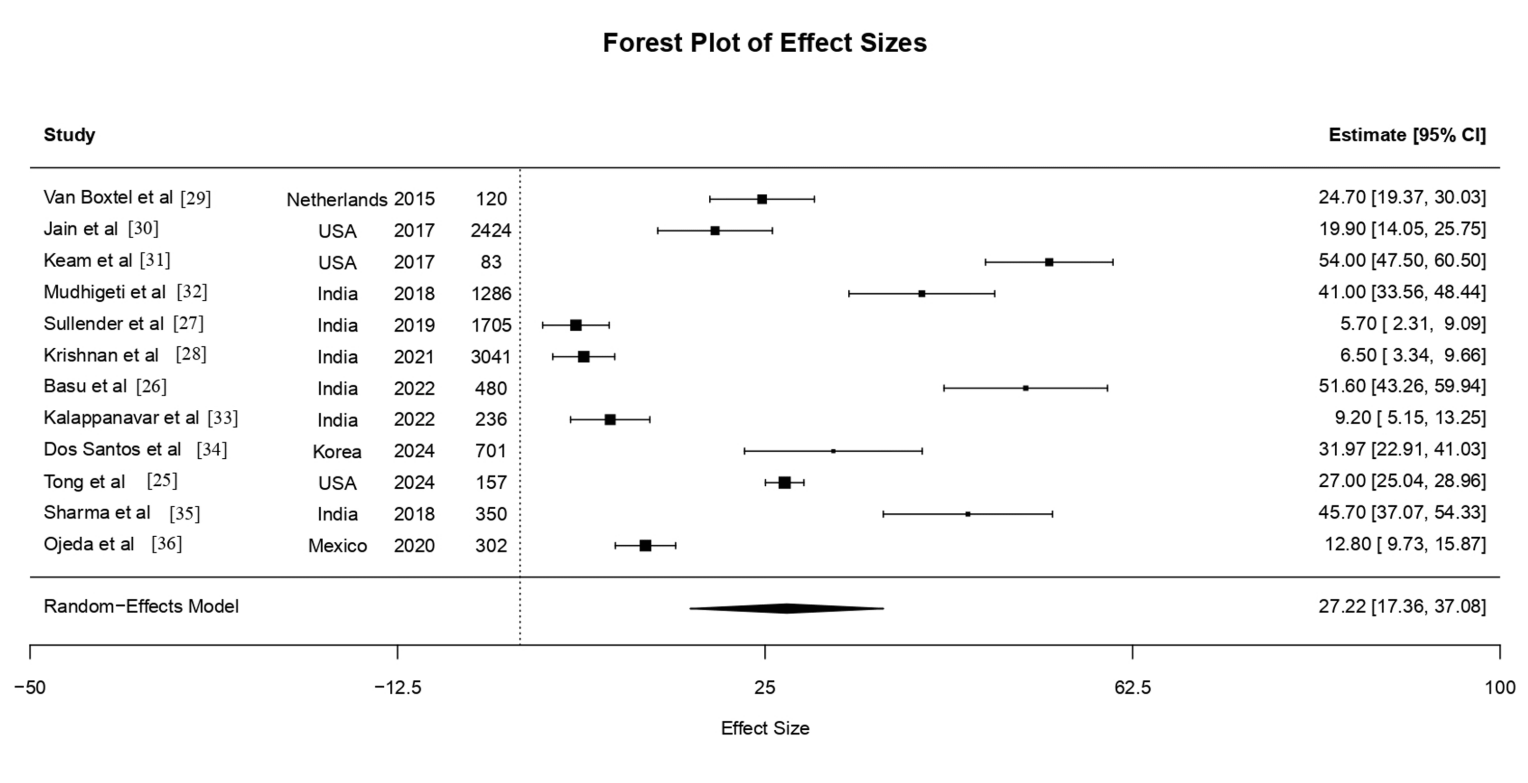
Forest Plot Depicting Pooled Vaccine Effectiveness From Included Studies

The forest plot illustrates the individual and pooled estimates of influenza VE across 12 studies included in the meta-analysis. Each horizontal line represents the 95% confidence interval for the VE reported in an individual study, plotted against a vertical line at zero effect. The point estimate of VE for each study is depicted as a square, with the size of the square proportional to the study’s weight in the analysis. The pooled estimate is represented by a diamond at the bottom of the plot (Figure 2).

The pooled VE across all studies was 27.22% (95% CI: 17.36%-37.08%), indicating a moderate protective effect of seasonal influenza vaccination. This estimate is lower than the 40-60% VE commonly reported in well-matched temperate seasons, suggesting that misalignment with peak influenza activity and waning immunity likely contributed to reduced effectiveness. Notably, substantial heterogeneity was observed among the studies, as reflected by the wide confidence intervals and statistical metrics (I² = 93.21%, p < 0.0001). Studies with higher VE generally corresponded to regions where vaccination timing was closely aligned with peak influenza activity [25, 26]. In contrast, lower VE was observed in settings where vaccination preceded the peak by several months or in populations with reduced immunogenic response [27, 28].

This figure supports the review’s conclusion that optimizing the timing of influenza vaccination campaigns based on regional seasonality may enhance overall VE.

**Figure 3:**
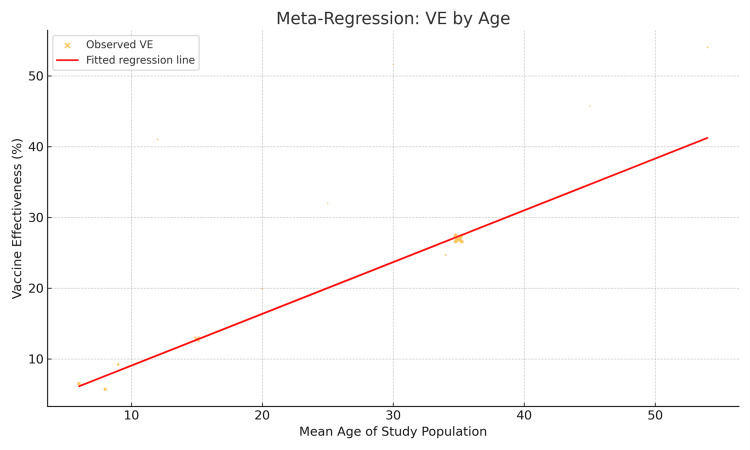
Meta-Regression Plot Exploring Sources of Heterogeneity in Vaccine Effectiveness.

The meta-regression plot illustrates the impact of selected moderator variables-primarily age group and vaccination timing-on the observed heterogeneity in VE across the 12 studies included in the meta-analysis (Figure 3).

The x-axis represents the covariates (e.g., mean age of study participants, region-specific vaccination timing in months prior to influenza peak), while the y-axis plots the reported effect size (VE) from each study. Each circle represents an individual study, with the size of the circle proportional to the inverse variance (i.e., study weight) in the regression model. The fitted regression line demonstrates the predicted VE based on the moderator variable, along with 95% confidence bands.

The regression model was performed using a random-effects meta-regression approach. It found that age was a significant predictor of VE (p < 0.05), with higher effectiveness generally seen in studies with younger populations (e.g., pediatric cohorts). This supports prioritizing boosters or high-dose/adjuvanted formulations for older adults and maintaining early campaigns for pediatric cohorts. 

Additionally, vaccination timing relative to the influenza peak also showed a strong association with VE (p < 0.01). Studies where vaccination closely preceded or matched the onset of peak influenza activity reported higher effectiveness.

Furthermore, the residual heterogeneity remained moderate, suggesting other unmeasured factors (e.g., comorbidities, vaccine type, circulating strain match) may contribute to outcome variability.

This supports the central conclusion of the review: that age and vaccination timing are important moderators of influenza VE. These findings further underscore the need for age-targeted strategies and regionally optimized vaccination calendars to enhance public health impact.

A summary of the 12 studies included in the meta-analysis, detailing study authors, publication year, country, participant age range or mean age, total sample size, and estimated VE with 95% confidence intervals. Studies represent diverse geographic regions and age groups, and were included based on eligibility for quantitative synthesis of influenza VE (Table 2).

**Table 2:** Summary of Meta-analyzed Studies Note: For India, studies spanned 2012–2023 and covered northern (winter peaks), central/eastern (monsoon), and southern (late monsoon) regions; national MoHFW campaign typically begins April–May

Study	Country	Year	Age range (Years)	Sample Size	Vaccine Effectiveness (%)	95% CI	References
Van Boxtel et al.	Netherlands	2015	18–49	120	24.7	10.2–39.2	[29]
Jain et al.	USA	2017	6–35	2424	19.9	2.5–37.3	[30]
Keam et al.	USA	2017	54	83	54.0	32.4–75.6	[31]
Mudhigeti et al.	India	2018	6–18	1286	41.0	12.8–69.2	[32]
Sullender et al.	India	2019	6–10	1705	5.7	-0.18–11.6	[27]
Krishnan et al	India	2021	2–10	3041	6.5	1.4–11.6	[28]
Basu et al.	India	2022	10–61	480	51.6	16.1–87.1	[26]
Kalappanavar et al.	India	2022	1–17	236	9.2	0.85–17.5	[33]
Dos Santos et al.	Korea	2024	1–50	701	31.97	-9.9–73.9	[34]
Tong et al.	USA	2024	18–51	157	27.0	25.0–29.0	[25]
Sharma et al.	India	2018	18–65	350	45.7	7.6–83.7	[35]
Ojeda et al.	Mexico	2020	6–17	302	12.8	8.0–17.6	[36]

Comparison of peak influenza activity periods and scheduled vaccination timings across different geographic regions. The table highlights the mismatch observed in some countries, such as India, where national vaccine campaigns precede the actual peak. Accurate alignment of vaccination timing with regional influenza seasonality may enhance overall VE and public health impact (Table 3).

**Table 3:** Regional Seasonality and Vaccination Timing. Peak timing sources: data are from individual studies or national reports – see citations next to each country.

Region/Country	Peak Influenza Season	Scheduled Vaccination Timing	Mismatch Observed
India [26]	July–September (monsoon); Jan–Mar (northern India)	April	Yes – vaccine precedes peak in many regions
USA [37]	December–February	September–October	No – vaccine aligns with peak
Netherlands [29]	December–February	September–October	No – vaccine aligns with peak
Korea [34]	December–January; secondary peak in April	October	Partial – secondary peak not addressed
Mexico [36]	November–March	October–November	No – vaccine aligns with peak

Discussion

This systematic review and meta-analysis provide a comprehensive evaluation of global influenza seasonality and the timing of vaccination campaigns, highlighting significant regional variations and their implications for VE. The findings underscore the necessity for region-specific vaccination strategies to optimize public health outcomes.​

Global Variations in Influenza Seasonality

Influenza seasonality exhibits considerable heterogeneity across different geographical regions. In temperate climates, such as North America and Europe, influenza activity typically peaks during the winter months, aligning with established vaccination schedules. Conversely, tropical and subtropical regions often experience multiple peaks or year-round circulation of influenza viruses, complicating the timing of vaccination campaigns. For instance, a study analyzing influenza patterns in tropical and subtropical Asian countries identified an early peak between June and October, with some countries experiencing a secondary peak in December to February [4]. This variability necessitates tailored vaccination strategies that consider local epidemiological data to ensure optimal protection.​

Impact of Vaccination Timing on Effectiveness

The alignment of vaccination timing with peak influenza activity is crucial for maximizing VE. Administering vaccines too early may result in waning immunity before the peak season, while delayed vaccination may leave populations unprotected during critical periods. U.S. modelling found that deferring vaccination from August to October could improve VE-provided coverage losses remain <10%-but warns of missed opportunities if too many defer, emphasizing a delicate balance in scheduling [37]. A systematic review highlighted that the effectiveness of influenza vaccines diminishes over time, emphasizing the importance of timely administration [38]. In India, for example, the national vaccination schedule typically commences in April; however, monsoon-associated influenza peaks occur between July and September (e.g., Delhi, Lucknow) [17], suggesting a potential misalignment that could compromise vaccine efficacy. Adjusting vaccination schedules to better coincide with regional influenza activity could enhance protective outcomes.

Age-Related Differences in Vaccine Effectiveness

VE varies across different age groups, with children often exhibiting higher responsiveness to influenza vaccines compared to older adults. Randomized trials in children aged 2-7 years showed LAIV efficacy of 69.2%-94.6% against matched strains, outperforming inactivated vaccines by ~52% during mismatched seasons, supporting LAIV use in pediatric immunization programs [39]. A meta-analysis revealed that fully vaccinated children aged 6 months to 17 years had a VE of approximately 62%, whereas partially vaccinated children had a VE of around 34% [40]. In contrast, older adults tend to have diminished immune responses, resulting in lower VE. In a US cohort, VE against A(H3N2) hospitalizations decreased by approximately 7.5% per month, with a more pronounced decline of 10.8% per month observed in adults aged 65 and older [5]. In China, a test-negative study of 15 212 outpatient and ER patients reported age-stratified VE of 32.2% (95% CI: 10.0-48.9%) in children <6 years, 48.2% (30.6-61.4%) in those aged 6-18, and 72.0% (43.6-86.1%) in adults 19-59, underscoring marked VE heterogeneity across age groups [41]. This disparity underscores the need for age-specific vaccination strategies, such as the use of high-dose or adjuvanted vaccines in the elderly to enhance immunogenicity.

Influence of Circulating Strains on Vaccine Performance

The match between vaccine strains and circulating influenza viruses significantly influences VE. Mismatches can lead to reduced vaccine performance, as observed during seasons dominated by the A(H3N2) subtype, which is known for its rapid antigenic drift. A study assessing the 2024-2025 influenza season in the United States reported VE estimates ranging from 36% to 54% among adults, with variations attributed to the predominance of different influenza A subtypes [42]. Continuous surveillance and timely updates to vaccine compositions are essential to maintain high levels of protection.

Waning Immunity and the Need for Booster Strategies

The durability of vaccine-induced immunity is a critical factor in influenza prevention. Evidence suggests that VE wanes over time, with significant reductions observed within months post-vaccination [38]. None of the 12 pooled studies directly modeled intra-season waning; this inference is drawn from broader literature (e.g., Doyon-Plourde et al. 2023). This waning effect poses challenges in regions with prolonged influenza activity or multiple peaks. Implementing booster vaccination strategies or adjusting the timing of initial doses could mitigate the impact of waning immunity and enhance overall protection.

Influenza Seasonality and Vaccination Timing in India

India's vast geographical diversity leads to significant variations in influenza seasonality across different regions. Studies have demonstrated that while northern regions like Srinagar experience influenza peaks during the winter months (January-March), other regions such as New Delhi observe peaks during the monsoon season (July-September) [17]. This variability complicates the establishment of a uniform national vaccination schedule. India’s Integrated Disease Surveillance Programme-IDSP and ICMR-NIV networks provide sentinel virological data, but coverage and timeliness vary, affecting schedule decisions.

A comprehensive analysis of influenza surveillance data from various Indian cities revealed three distinct seasonal patterns:

Temperate Seasonality: Cities like Srinagar exhibit influenza peaks during the winter months, aligning with patterns observed in temperate regions.

Monsoon-Associated Peaks: Cities such as Delhi, Lucknow, Pune, and Kolkata experience peaks during the monsoon season (July-September).

Late Monsoon Peaks: Southern cities like Chennai and Vellore show influenza activity peaking during the late monsoon months (October-November) [43].

These findings underscore the importance of region-specific vaccination strategies. For instance, initiating vaccination campaigns in April-June would be more effective for regions with monsoon-associated peaks, while September-October would be optimal for areas with winter peaks [44]. Implementation must account for cold-chain constraints, procurement cycles, and campaign synchrony with other national programs, which complicate shifting schedules.

Vaccine Effectiveness in the Indian Context

The effectiveness of influenza vaccines in India has been evaluated through various studies. A case-control study conducted in Pune assessed the effectiveness of an LAIV against the A(H1N1) pdm09 strain. The study reported an adjusted VE of 76% (95% CI: 42.1-89.7), indicating a high level of protection [45].

Another randomized controlled trial in rural India compared the efficacy of LAIV and inactivated influenza vaccine (IIV) among children aged 2-10 years. The study found that both vaccines were safe and moderately efficacious, with VE ranging from 40% to 59% across different years [28].

These studies highlight the potential of both LAIV and IIV in providing substantial protection against influenza in the Indian population, emphasizing the need for their inclusion in national immunization programs.

Policy Implications and Recommendations

Given the regional variations in influenza seasonality and the demonstrated effectiveness of vaccines, it is imperative for public health authorities in India to adopt flexible vaccination strategies tailored to local epidemiological patterns. For instance, Bangladesh aligned campaigns closer to southern-hemisphere recommendations, improving strain match and coverage in peak months, as reported by Berry et al. 2022 [15]. The Indian Association of Preventive and Social Medicine (IAPSM) recommends initiating vaccination campaigns in April-June for regions with monsoon peaks and in September-October for areas with winter peaks [44].

Implementing such region-specific vaccination schedules would enhance the overall effectiveness of influenza prevention efforts, reduce morbidity and mortality, and optimize resource utilization. Operationalizing regional calendars requires continuous sentinel surveillance, rapid data sharing (weekly ILI/virological reports), and forecasting capacity within MoHFW/ICMR networks.

## Conclusions

In summary, this systematic review and meta-analysis demonstrates that influenza’s seasonality is highly variable across the globe, which in turn affects vaccine performance. A global analysis of influenza patterns shows that a one-size-fits-all vaccination schedule is ineffective because flu seasons vary significantly worldwide, especially in tropical and subtropical zones. The study concludes that tailoring vaccination timing to specific regional seasons could significantly boost VE. The authors recommend that public health officials adopt flexible, region-specific vaccination calendars and conduct further research to optimize these strategies.
